# Supplementary material for: Identification of photoperiod-induced specific miRNAs in the adrenal glands of Sunite sheep (Ovis aries)
Source: Front Vet Sci. 2022 Jul 22;9:888207. doi: 10.3389/fvets.2022.888207 (PMC9354845; doi:10.3389/fvets.2022.888207)
Supplement: Supplementary file 1 [file Data_Sheet_1.ZIP › Supplementary file/miRDeep2 file.zip!/miRDeep2 file/Know_Structure/SPLP42A1/oar-mir-487b.pdf]

miRBase precursor : oar-mir-487b
Total read count : 3814
oar-miR-487b-5p read count : 80
oar-miR-487b-3p read count : 3729
remaining reads : 5

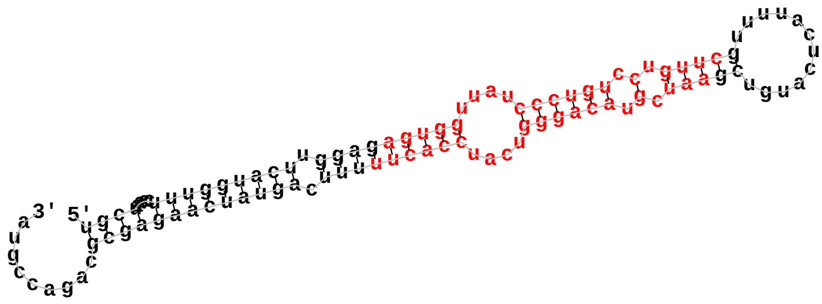

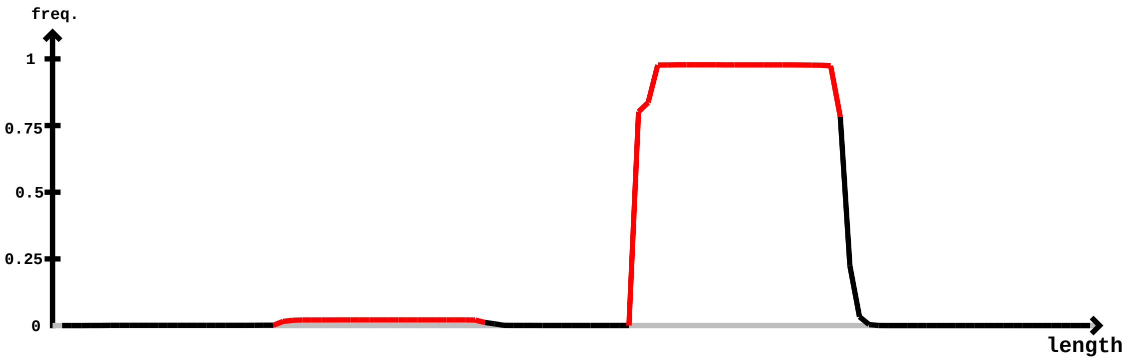

oar-miR-487b-3p

oar-miR-487b-5p

5'- ugcuaaccuuugguacuuggagagugguuaucccuguccuguucguuuuacucaugucgaaucguacagggucauccacuuuuucaguaucaagagcgcagaccgua -3'	exp		
.(((....(((((((((.(((((((((....((((((.(.(((((............))))).).))))))....))))))))).))))))))))))..........	reads	mm	sample
..cuaaccuuugguacuuggag...	1	0	seq
....aaccuuugguacuuggaga..	2	0	seq
...................gagagugguuaucccuguccuguu..	1	0	seq
....................agagugguuaucccuguccuguu..	1	0	seq
......................agugguuaucccuguccugu...	1	0	seq
......................agugguuaucccuguccuguu..	29	0	seq
......................agugguuaucccuguccuguuc...	15	0	seq
......................agugguuaucccuguccuguucg..	10	0	seq
......................agugguuaucccuguccuguucgu...	1	0	seq
.......................gugguuaucccuguccugu...	1	0	seq
.......................gugguuaucccuguccuguu..	1	0	seq
.......................gugguuaucccuguccuguuc...	5	0	seq
.......................gugguuaucccuguccuguucg..	10	0	seq
........................ugguuaucccuguccuguu..	2	0	seq
........................ugguuaucccuguccuguucg..	3	0	seq
............................uaucccuguccuguucguuuu..	1	0	seq
....uuuuacucaugucgaaucguaca.......................................	1	0	seq
....aaucguacagggucauc...............................	2	0	seq
....aaucguacagggucaucc..............................	2	0	seq
....aaucguacagggucaucca.............................	2	0	seq
....aaucguacagggucauccac............................	7	0	seq
....aaucguacagggucauccacu...........................	715	0	seq
....aaucguacagggucauccacuu..........................	2064	0	seq
....aaucguacagggucauccacuuu.........................	265	0	seq
....aucguacagggucauccacu...........................	9	0	seq
....aucguacagggucauccacuu..........................	33	0	seq
....aucguacagggucauccacuuu.........................	77	0	seq
....aucguacagggucauccacuuuu........................	13	0	seq
....ucguacagggucauccacu...........................	6	0	seq
....ucguacagggucauccacuu..........................	31	0	seq
....ucguacagggucauccacuuu.........................	387	0	seq
....ucguacagggucauccacuuuu........................	99	0	seq
....ucguacagggucauccacuuuuu.......................	13	0	seq
....ucguacagggucauccacuuuuuc......................	1	0	seq

```
                                                                oar-miR-487b-3p
                      oar-miR-487b-5p
ugcuaaccuuugguacuuggagagugguuaucccuguccuguucguuuuacucaugucgaaucguacagggucauccacuuuuucaguaucaagagcgcagaccgua

...............................................................guacagggucauccacuu..........................    3    0    seq
```
